# The Effect of Time-Restricted Eating on Insulin Levels and Insulin Sensitivity in Patients with Polycystic Ovarian Syndrome: A Systematic Review

**DOI:** 10.1155/2022/2830545

**Published:** 2022-09-16

**Authors:** R. Floyd, R. Gryson, D. Mockler, J. Gibney, S. N. Duggan, L. A. Behan

**Affiliations:** ^1^Department of Endocrinology, Robert Graves Institute of Endocrinology, Tallaght University Hospital, Dublin, Ireland; ^2^Department of Clinical Medicine, School of Medicine, Trinity College Dublin, Dublin, Ireland; ^3^Department of Obstetrics & Gynaecology, Rotunda Hospital, Dublin, Ireland; ^4^John Stearne Medical Library, Trinity College Dublin, Trinity Centre for Health Sciences, Dublin, Ireland; ^5^Department of Surgery, School of Medicine, Trinity College Dublin, Dublin, Ireland

## Abstract

**Results:**

2662 papers were identified with 37 selected for full-text review and one paper meeting criteria for inclusion. Ramadan fasting was the only time-restricted eating regimen trialled in this population with no strong evidence of a significant effect on insulin levels.

**Conclusion:**

As the systematic review retrieved only one study investigating time-restricted eating to reduce insulin in patients with PCOS, there is no evidence to suggest that this intervention is effective. From the narrative review, based on studies in other patient groups, time-restricted eating could improve insulin resistance in those with PCOS; however, well-designed studies are required before this intervention can be recommended.

## 1. Introduction

Polycystic ovarian syndrome (PCOS) affects up to 10% of premenopausal women and is characterised by multiple ovarian cysts, hyperandrogenism causing hirsutism, hyperinsulinemia, insulin resistance, obesity, infertility, and mood disturbances [1]. Affected women have an increased risk of developing endometrial cancer, cardiovascular disease, hypercholesterolaemia, and type 2 diabetes mellitus meaning that early intervention and management are of crucial importance [2]. Both pharmacological [2–17] and nonpharmacological therapies (based around weight loss, diet, and exercise) are employed, the latter being the foundation of PCOS management.

The majority of women with PCOS have insulin resistance, independent of weight [18–20]. This is often subclinical and may not be reflected in routine biochemical tests [21]. Despite this, hyperinsulinemia and abnormalities in glucose haemostasis lead to cardiovascular and systemic inflammatory changes [22]. Women with PCOS are more likely to have postprandial hyperglycaemia owing to the peripheral insulin resistance rather than fasting hyperglycaemia due to intact endogenous glucose production mechanism [23]. Overall data suggests that 1 in 3 women with PCOS have impaired glucose tolerance and 1 in 10 have type 2 diabetes, with the majority (60%) being overweight or obese [1].

**Figure 1:**
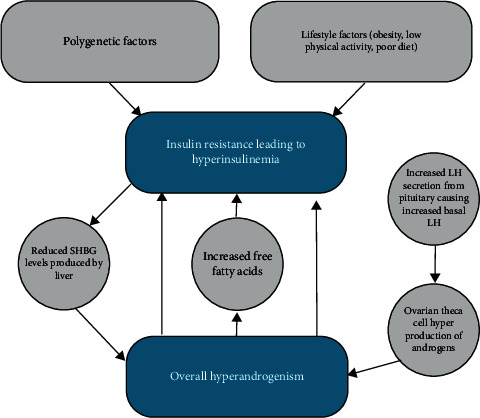
Hyperinsulinemia downstream effects on ovarian theca cell hyperproduction of androgens and subsequent pituitary feedback causing increased basal luteinizing hormone. Hyperandrogenism results in reduced SHBG (sex hormone binding globulin) (often used clinically for assessment of insulin resistance in PCOS).

Hyperinsulinemia is linked with persistent hyperandrogenism, as well as its clinical manifestations, by directly driving excessive androgen production (Figure 1). Due to the relationship between increased insulin levels and androgens, the treatment of hyperinsulinemia with pharmacological and nonpharmacological approaches is essential, notwithstanding the preclinical effects of high insulin levels and predisposition to diabetes. Insulin resistance has also been implicated in cravings for carbohydrates and subsequently overeating, binge pattern eating, and weight gain [24, 25]. This has the potential to have confounding effects on the overall risk of hyperinsulinemia, subsequent diabetes, and cardiovascular disease. Obesity in PCOS is a key driver of deranged cardiometabolic parameters including insulin resistance, hyperandrogenism, and dyslipidaemia. Obesity exacerbates these conditions compared to women with PCOS who have a normal BMI. In particular, central obesity is associated with increased severity and worsening of insulin resistance [26].

Dietary interventions in PCOS are varied with many diets being unsustainable and most largely ineffective. Dietary regimens including energy (calorie) restriction [27, 28], Mediterranean diet [29], low carbohydrate diet [30], dietary approaches to stop hypertension (DASH) diet [31], pulse-based diet [32], low-starch diet [33], low-dairy diet [34], ketogenic diet [35], and low-glycaemic-index diet [36] have been trialled in patients with PCOS. Current dietary recommendations for patients with PCOS include regular eating, avoidance of refined sugars, low-glycaemic-index foods, and carbohydrate-rich diets, with calcium/vitamin D supplementation [37]. While the restriction of calories and an increase in exercise improve insulin sensitivity in overweight patients with PCOS, sustained calorie-restricted diets over a prolonged period of time are difficult to sustain [38]. More recently, the effect of time-restricted eating (also termed intermittent fasting) in PCOS has been examined [39]. In contrast to diets based specifically on food restriction, time-restricted eating, where patients are asked to consume all energy within a restricted daily time period, appears to offer more sustainable weight loss and cardiometabolic changes and may be more acceptable as a permanent lifestyle change [38].

Time-restricted eating involves a period of fasting, thereby allowing a decrease in insulin levels, an improvement in insulin sensitivity, and an improvement in glucose regulation. The short-term putative benefits of time-restricted eating include increased cell metabolic and oxidative stress resistance [40]. A period of daily fasting depletes liver glycogen stores and switches energy sources to fatty acid and ketones. This bioenergetic challenge activates signalling pathways that strengthen mitochondrial function and stress resistance and upregulate autophagy of damaged molecules adopting a stress resistance mode [40]. This reduces insulin signalling and overall protein synthesis. During refeeding following the daily “fast,” glucose levels rise and ketones are cleared with increased protein synthesis allowing growth and repair allowing more efficient cellular performance, leading to cellular resilience and disease resistance as a long-term adaptation [38]. A time-restricted eating regimen where evening food intake is restricted reportedly improves postprandial insulin and glucose handling due to alignment of circadian rhythm with diurnal food intake [41]. Several variations of time-restricted eating and terms are described in Table 1.

Fasting-induced fuel switching has the potential to overcome the issues seen in patients with PCOS, as seen in other populations with underlying insulin resistance, including metabolic syndrome, obesity, and type 2 diabetes [42–44]. Time-restricted eating represents a novel solution to aid in the control of insulin resistance in patients with PCOS in combination with already established nonpharmacological and pharmacological managements. This review aims to conduct a systematic review and meta-analysis of intervention studies on time-restricted eating in patients with PCOS and to conduct a further narrative review of the related literature to guide further research.

## 2. Methods

This systematic review followed PRISMA reporting guidelines [45]. This review was registered with the PROSPERO registry under registration number CRD42021267268.

### 2.1. Review Question

The PICO model was employed to expand the return from the review and applicability of data collected.

- Population: Females above the age of eighteen years, diagnosed with polycystic ovarian syndrome using Rotterdam criteria.
- Intervention: Any time-restricted feeding regimen, alone or in combination (pharmacological therapy, exercise, and weight loss). To include 16 : 8 method, 18 : 6 method, 5 : 2 diet, alternate day fasting, intermittent fasting, Ramadan or other religious fasting methods deemed suitable.
- Comparator: Comparison to usual ad libitum diet or no dietary or fasting intervention, standard treatment-as-usual, and pharmacological therapy.
- Outcomes: Metabolic parameters which represent effect on insulin levels, C peptide concentrations, glucagon, insulin-like growth factor 1 (IGF-1), glycated haemoglobin (HbA1c), homeostatic model assessment for insulin resistance (HOMA-IR), fasting blood glucose, sex hormone binding globulin (SHBG), or oral glucose tolerance test (OGTT).

### 2.2. Search Strategy and Included Study Selection

**Figure 2:**
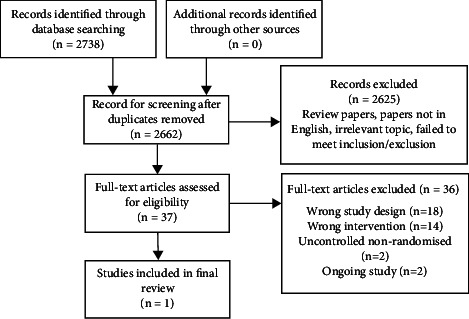
PRISMA (Preferred Reporting Items for Systematic Reviews and Meta-Analyses) diagram of systematic review search.

A preliminary scoping review was conducted to identify the nature and extent of the research available. A systematic search strategy was constructed with oversight of a medical librarian DM. Five search strategies were created for Medline, CINAHL (Cumulative Index to Nursing and Allied Health Literature), EMBASE, WOS (Web of Science), and Cochrane Library, the details of which are found in Appendix A. The five databases searched yielded 6340 potential papers: Medline (1737), CINAHL (339), EMBASE (1909), WOS (2086), and Cochrane Library (268). After removal of duplicates, 2739 papers remained. No further studies were identified by hand-reviewing citation lists of eligible studies, previous reviews, and field expert publications. 37 papers underwent full-text review and 1 met inclusion criteria (Figure 2). Keywords used for searches included “polycystic ovarian syndrome or PCOS,” “time-restricted feeding or TRF,” “insulin levels,” “metabolic changes,” “endocrine changes,” “diet,” and “Ramadan.” Full search details are available in Appendix A. Results were combined from the five databases and papers were selected for full-text review after dual-screening by two independent reviewers (R.F. and R.G.) who made independent decisions on inclusion. All conflicting decisions were resolved by a senior reviewer (S.D.). Further manual searches of the reference lists of all relevant papers were carried out for additional relevant papers. Studies were excluded based on incorrect study design, incorrect intervention, incorrect setting, incorrect population, failure to meet inclusion criteria, duplicate not detected, ongoing studies, still recruiting, noncontrolled trials, and different outcome measured with full details outlined in Table 2. The following data was extracted from each paper: study design, patients lost to follow-up, completion of intention-to-treat analysis, group allocation method, participant numbers, age range, study population, country, baseline preintervention weight and insulin levels, intervention type, comparison, and outcome assessment.

### 2.3. Inclusion/Exclusion Criteria

- *Timeline*: Published before 10th of May 2021 when final searches were run.
- *Patients*: Female patients with a diagnosis of polycystic ovarian syndrome by Rotterdam criteria.
- *Inclusion*: All study designs considered which were published in peer-reviewed medical journals. Studies involving time-restricted eating, Ramadan fasting, and diets with any fasting regimen were considered, in addition to studies where time-restricted eating/intermittent fasting combined with concurrent medication use (combined oral contraceptive pill (COCP), metformin, spironolactone, etc.). Studies must have baseline and postintervention measures of outcomes. Pilot studies were assessed for inclusion if clear outcome effects were detailed. Only studies involving humans were included.
- *Exclusion*: Conference papers, unpublished reports, letters to the editor, papers not in English were excluded. Studies considering administration of supplements, studies without a control group, studies without a baseline compared to postintervention measures, and nonhuman, preclinical, and animal studies were also excluded.

### 2.4. Assessment of Results

Results of included studies were extracted by two independent reviewers (R.F. and R.G.). Study characteristics were collected including details of participants, study methodology, intervention details, and effect on insulin and other metabolic parameters. Analysis to assess study quality and risk of bias was conducted in accordance with Cochrane Handbook for Systematic Reviews of Interventions.

### 2.5. Analysis/Data Synthesis

The studies included in the review were inherently heterogeneous due to varying interventions and outcomes. As a result, a descriptive presentation of the studies was used without pooling of outcomes for analysis.

## 3. Results

### 3.1. Populations and Interventions

The one study included was a nonrandomised controlled study with a Ramadan fasting regimen. This study recruited women with a PCOS diagnosis. The details of the study, population characteristics, and outcomes were extracted and reviewed.

### 3.2. Outcomes

Due to the lack of studies identified for inclusion and heterogeneity of study designs, it was not possible to conduct a meta-analysis.

#### 3.2.1. Ramadan Fasting

One small study was identified which looked at 40 women with PCOS, 20 of whom completed Ramadan fasting for a mean of 26 days and were compared to a nonfasting control group. Details of the length of fasting times were not included but rather details of eating patterns before sunrise, after sunset, and between sunset and sunrise. There were no significant differences in levels of beta-endorphin (*p*=0.543), insulin (*p*=0.818), FSH, LH, testosterone, or adrenaline after undergoing Ramadan fasting. Significant effects of Ramadan fasting on reducing cortisol (*p*=0.049) and noradrenaline levels (*p*=0.047) were shown. Overall there was no benefit of Ramadan fasting shown in this study on insulin levels or glucose haemostasis [46].

### 3.3. Risk of Bias in Included Study

The risk of bias was categorised into low, high, or unclear risk for selection bias, reporting bias, performance bias, detection bias, and attrition bias. The risk of bias assessment was conducted by two independent reviewers (RF and RG). The overall risk of bias was deemed to be low.

## 4. Discussion

A systematic review of the literature found that the effects of time-restricted eating on insulin levels in patients with PCOS has not been investigated to date, and therefore there is no evidence to suggest that this intervention would be effective in reducing insulin in patients with PCOS. The systematic review retrieved just one study [46] which investigated on the effect of Ramadan fasting on insulin levels in patients with PCOS. This study showed no effect of Ramadan fasting on insulin and glucose homeostasis in patients with PCOS versus controls.

### 4.1. Narrative Review

The systematic review had excluded two studies investigating the effects of time-restricted eating as they were noncontrolled. The first of these [39] investigated the effect of a 16 : 8 time-restricted eating on anthropometric parameters, sex hormones, insulin resistance parameters, inflammatory markers, lipids, menstrual cycle, and eating behaviours in 18 women with PCOS. Participants completed a 16-hour fast daily for five weeks, preceded by a one-week baseline weight stabilisation period. Three of the 18 participants (16.7%) dropped out of the trial. Time-restricted eating (16 : 8) reduced fasting insulin levels (*p*=0.017), area under the curve for insulin (AUCIns) (*p*=0.007), ratio of AUCIns/AUCGlu (*p*=0.001), HOMA-IR (*p*=0.0025), SHBG (*p* < 0.001), body weight (*p* < 0.001), BMI (*p* < 0.001), body fat mass (*p* < 0.001), percentage of free androgen index (*p*=0.001), CRP (*p*=0.040), ALT (*p*=0.027), and IGF-1 (*p*=0.006). There were no significant differences in fasting glucose, area under the curve for glucose (AUCGlu), lipid profiles, and gonadal parameters including luteinizing hormone (LH), follicular stimulating hormone (FSH), and LH/FSH ratio or total testosterone. Overall, this study suggested that 16 : 8 time-restricted regimen improved parameters of glucose haemostasis and weight [39] in women with PCOS.

The second study [47] investigated the effects of Ramadan fasting in 27 women who fasted for a mean period of 16.5 hours a day for 29 days. There were no significant differences in weight (*p*=0.439), BMI (*p*=0.646), fasting blood glucose (*p*=0.183), insulin (*p*=0.474), HOMA-IR (*p*=0.364), HOMA-B (*p*=0.736), QUICKI (*p*=0.308), or lipid profiles. There were significant decreases in C-reactive protein levels (*p*=0.072), nitric oxide (NO) levels (*p*=0.003), and total glutathione levels (*p*=0.011). This study corroborated the systematic review finding that Ramadan fasting exerted no benefit on parameters of glucose haemostasis but had modest benefits on markers of inflammation and cardiovascular health [47]. Overall, considering both controlled and noncontrolled studies mentioned above, Ramadan fasting does not appear to have an effect on insulin levels or glucose haemostasis in individuals with PCOS.

There is some evidence from small studies in other patient groups that Ramadan fasting may have positive benefits on insulin levels and glucose haemostasis. Ramadan improved HbA1c levels in 29 patients with type 2 diabetes [48] and was associated with a decrease in glucose levels in 80 healthy subjects [49]. There appears to be modest benefits on hormonal markers of stress (cortisol and noradrenaline levels) [46], inflammation (C reactive protein or CRP levels), and cardiovascular health (NO and glutathione (GSH) levels) [47]. The underlying mechanism of these benefits remains to be defined and elucidated.

### 4.2. Weight Loss: A Potential Confounder?

In studies on time-restricted eating reporting weight loss [39], it is difficult to establish whether the improvements in glucose homeostasis are secondary to the concomitant weight loss rather than being a consequence of time-restricted eating. The majority of studies that cite therapeutic benefits of fasting regimens in various patient populations also report weight loss, with most beneficial cardiometabolic effects as a result of the latter [50–53]. Time-restricted eating may result in a reduction of energy intake from baseline levels. It is difficult to remove this confounding factor when designing studies on time-restricted eating, as asking patients to restrict their eating to a limited period might result in reduced calorie intake and subsequent weight loss. However, arguably this is an important outcome in itself. While “permanent” energy-restricted diets will result in weight loss, eating fewer calories appears to be virtually impossible in the long term. If time-restricted eating results in a sustainable, long-term reduction in energy intake due to its effect on appetite or satiety, then this will be of clear value for a myriad of patient types. Animal studies have shown that time-restricted feeding, without reduction of calories, shows protection against hyperinsulinemia and improves overall hepatic glucose metabolism [54–59]. Animal studies have also shown that time-restricted feeding of a high fat, diary, and sugary drinks containing western diet can have positive effect on metabolic effects without weight loss [58]. A small study in prediabetic men has shown benefits of a 6-hour time-restricted eating period on cardiometabolic profiles, including reduced blood pressure, reduced oxidative stress, and reduced appetite as well as increased insulin sensitivity and *β* cell responsiveness, independent of weight loss [60]. However, whether or not the benefits of time-restricted eating are independent of weight loss, particularly in patients with PCOS, is not known and has been poorly researched. Ideally, randomised human studies on patients with insulin resistance (including type 2 diabetes, metabolic syndrome, or PCOS) should be carried out. A study design comparing time-restricted eating with continuous eating, with both arms receiving isocaloric, meal frequency-matched diets, would be of value.

Li et al. observed significant results in a short, time-restricted eating regimen suggesting that improvements in hyperinsulinemia and insulin resistance can be seen without energy restriction [39]. This is contrary to a review reporting isocaloric time-restricted feeding which had a greater benefit in reducing fasting insulin and insulin resistance than ad libitum time-restricted feeding [61]. Li et al.'s study lacks power being a nonrandomised noncontrolled intervention study on a small number of participants. The short duration of the intervention was also a limitation of these studies, similar to the Ramadan studies. It is important to assess these over time to assess for compliance with fasting regimens.

### 4.3. Optimum Timeline

The optimum length of a time-restricted eating intervention to have a significant effect on insulin levels is undecided. Initially switching from standard dietary pattern to intermittent fasting, hunger, irritability, and reduced concentration ability while adapting to the new dietary regimen is expected [40]. These initial effects usually dissipate within one month potentially making this a reasonable minimum time for an intervention.

### 4.4. Low-Calorie Drinks

Li et al. [39] allowed the consumption of low-calorie sweetened drinks during the fasted period in their study on time-restricted eating. Although artificial sweeteners reportedly have negligible effects on insulin levels [62, 63], such ingredients (sucralose, aspartame, and saccharin) may adversely affect the gut microbiome which may indirectly impair glucose haemostasis, causing insulin resistance and contributing to metabolic disease [64]. While artificial sweetened and low- or zero-calorie diets facilitate a reduction in food energy content, promote satiety, and might ultimately reduce weight, their effects on insulin levels during a period of fasting should be considered. The effects of other ingredients present in artificially flavoured beverages have unknown effects on glucose and insulin levels during an otherwise fasted period. Arguably, a true fasted period should not include sweet-tasting beverages.

### 4.5. How the Intervention Might Work

Time-restricted eating reportedly causes a shift in fuel source from glucose to fatty acids during the fasting period [38]. Typically twelve hours into a fast, there is depletion of liver glycogen stores and fatty acids are mobilized and released as the main energy fuel [38]. By aligning meal times with the light-dark cycle, energy intake, weight control, and glucose and insulin levels may be optimised [65]. Shift workers who oppose the latter concept have higher rates of cardiometabolic dysfunction [65]. Time-restricted eating may also positively affect the gut microbiome [66]. The gut microbiome is heavily influenced by diet, as well as circadian rhythm, with time-restricted eating shown to improve intestinal bacteria microenvironment with increasingly favourable microbial profile. The downstream effects of this include favourable metabolic regulation and reduction in inflammation [67]. Time-restricted eating in non-PCOS patients resulted in weight loss, with meta-analysis showing a weighted mean difference of 2 kg in observational studies and 0.4 kg in randomised controlled trials analysed, but with limited improvements in insulin levels, glucose haemostasis, and lipid profiles [67]. Compliance is similar for patients following a time-restricted eating regimen compared to traditional calorie-restricted dietary regimens [50].

Typical ad libitum eating (with short fasting periods) might perturb normal glucose metabolism. Eating habits have largely changed with modern lifestyle. A longitudinal study of dietary habit changes over a 40-year timespan in USA showed higher daily energy intake, later breakfast and lunch times, and shorter time between dinner and a postdinner snack, with a significant increase in snacking among women in particular [68]. There are few, if any, human studies on continuous feeding and its effect on insulin levels. It could be hypothesised that long eating windows might increase the risk of insulin resistance and metabolic disorders. In animal studies comparing continuous ad libitum with time-restricted feeding, ad libitum feeding leads to increased rates of obesity, cancer, renal and cardiovascular disease, and decreased overall survival [69].

**Figure 3:**
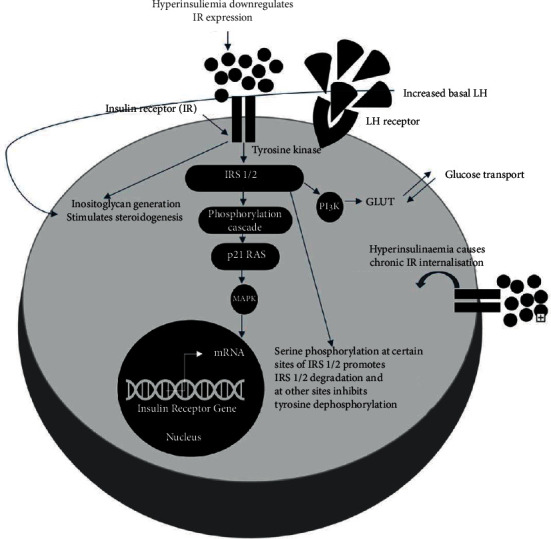
Illustration of the effect of hyperinsulinemia on insulin receptors and stimulation of steroidogenesis caused by hyperinsulinemia and increased LH (luteinizing hormone) levels. Insulin receptor defects are due to serine phosphorylation of the insulin receptor and IRS-1 (insulin receptor substrate 1) secondary to intracellular serine kinases. This results in reduced PI3K (phosphoinositide 3-kinase) downstream activity after insulin mediated activation and resistance to the metabolic actions of insulin. Activation of kinases in ERK/MAPK (extracellular signal-regulated kinases/mitogen-activated protein kinases) mitogenic pathway in PCOS (polycystic ovarian syndrome) causes inhibitory serine phosphorylation of IRS-1 in skeletal muscle in patients with PCOS as demonstrated here. These defects in the insulin receptor gene exist in patients with PCOS with extreme insulin resistance although insulin receptor numbers and affinity are similar to those in the patient without insulin receptor defects. Steroidogenesis is stimulated by both hyperinsulinemia causing inositolglycan generation and increased basal LH secreted from the anterior pituitary in response, also demonstrated below.

On a molecular level, the biochemical effects of time-restricted eating in PCOS are thought to include the following:

1. Increased recruitment of insulin receptors, internalised by the effects of persistent hyperinsulinemia as demonstrated in Figure 3.
2. Upregulation of insulin receptor expression which is downregulated by hyperinsulinemia.
3. Lowering insulin requirements with fasting. Fasting overcomes the issues seen in patients with PCOS including saturation of defected insulin receptors, beta cell dysfunction, and reduced hepatic insulin clearance [70–72].
4. Reduced leptin levels and increased adiponectin with an overall improved balance improving insulin resistance [42–44, 73, 74].

**Figure 4:**
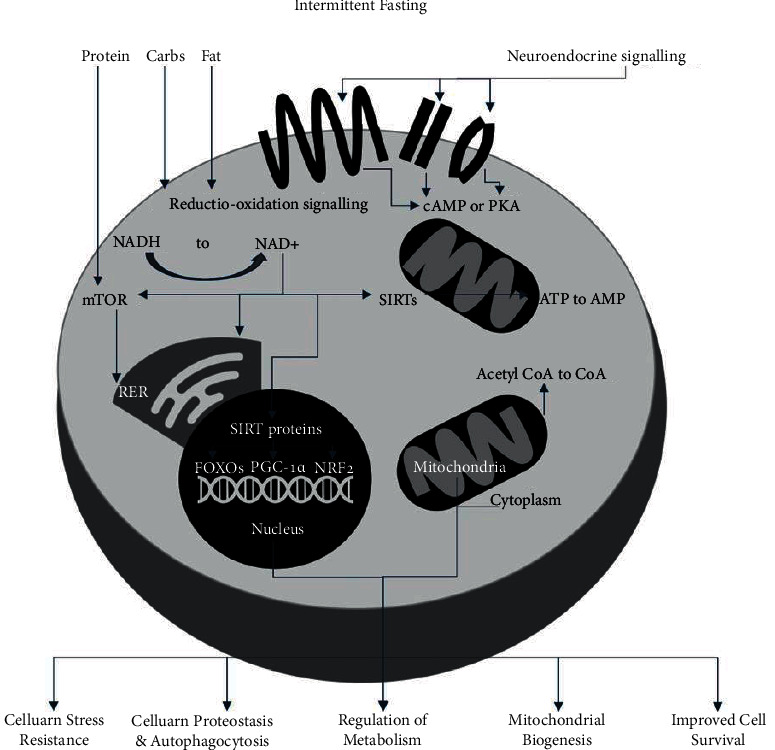
Cellular metabolic pathways and responses to intermittent fasting showing downstream cascade effects of fasting and resulting beneficial outcomes {NAD+ (nicotinamide adenine dinucleotide); transcription factors FOXOs (forkhead box Os), PGC-1*α* (proliferator-activated receptor *γ* coactivator 1*α*), and NRF2 (nuclear factor erythroid 2-related factor 2); kinases AMPK (AMP kinase) and cAMP cyclic AMP; and deacetylases sirtuins (SIRTs); mammalian target of rapamycin (mTOR); insulin-like growth factor 1 (IGF-1)}.

On a cellular level, time-restricted eating reportedly contributes to the fluctuating ratios of NAD+ to NADH, ATP to AMP, and acetyl CoA to CoA. These result in downstream activation of proteins including transcription factors (FOXOs, PGC-1*α*, and NRF2), AMP kinase, and deacetylases such as sirtuins (SIRTs) [38, 40], as illustrated in Figure 4. These might help to regulate cellular basic function and improve cellular stress resistance. Acetyl CoA and NAD+ serve as cofactors for these SIRTs epigenetic modifiers [38, 40]. SIRTs deacetylate transcription factors mentioned and promote gene expression, aiding with cellular stress resistance (Figure 4).

Fasting reportedly results in downregulation of the insulin-IGF-1 signalling pathway and reduced circulating amino acids causing reduced mTOR activity with subsequent inhibition of protein synthesis. All of the above processes allow activation of cellular repair and maintenance processes, stress resistance and mitochondrial biogenesis, cellular proteostasis, and autophagy with overall improved cell survival. The AMP to ATP ratio is increased by fasting, activating AMPK, which triggers repair and halts anabolic processes [40].

### 4.6. Suggested Future Research

A prospective randomised intervention study with crossover design would be best to determine the effect of a time-restricted eating pattern on insulin levels, androgens, fertility, and satiety. We suggest that a controlled feasibility study should be conducted to investigate the feasibility, safety, compliance, and acceptance of time-restricted eating in patients with PCOS, as well as its effects on insulin, androgens, and satiety, and have registered a study to investigate these outcomes (NCT05126199).

## 5. Conclusions

The systematic review found no studies investigating the effect of time-restricted eating on insulin levels in patients with PCOS, with the exception of one study on Ramadan fasting which showed no effect. Although a narrative review discussed an uncontrolled study which showed improvement in glucose homeostasis, weight, and androgens with 16 : 8 time-restricted eating in PCOS, overall we conclude that there is insufficient evidence that time-restricted eating works to reduce insulin levels in PCOS, and, pending further studies, the intervention should not be recommended in this group.

## Figures and Tables

**Table 1:** Summary of typical intermittent fasting/time-restricted eating regimens.

Regimen	Description
Intermittent fasting/time-restricted eating	Most commonly involves fasting for 16–18 hours with an eating window of 6–8 hours, leveraging the natural circadian rhythm. Plain water and unsweetened fluids (black plain tea/coffee) are allowed
Time-restricted feeding	Restriction of caloric intake to specific time periods of the day, typically 8–12 hours during daytime hours. Term typically used in animal studies
Alternate day complete fasting	No calorie intake on fasting days, followed by a day of ad lib intake (eating to satiety)
Alternate day modified fasting	Restricted calorie intake on “fasting” days (<25% daily energy requirements), alternated with days of ad lib intake (eating to satiety)
Periodic fasting	1-2 fasting days/week and 5-6 days of normal caloric intake
5 : 2	
6 : 1	
Ramadan fasting	Fasting from dawn until sunset followed by ad lib calorie intake after sunset to before dawn. Similar to time-restricted eating but conflicts with circadian rhythm
	No water or fluids during fasting

**Table 2:** Excluded studies after full text review.

Study ID number (Covidence) and author	Reason for exclusion
1. #2560 Agowska 2021	Wrong intervention
2. #169 Altieri 2013	Wrong study design
3. #2263 Anderson 1995	Wrong intervention
4. #582 Armutcu 2019	Wrong study design
5. #2348 Asemi 2014	Wrong intervention
6. #419 Asemi 2015	Nonrandomised and without control group
7. #2213 Chiofalo 2017	Wrong study design
8. #1889 El-Bandrawy 2016	Wrong intervention
9. #2019 Farshchi 2007	Wrong study design
10. #2085 Fransk 1991	Wrong study design
11. #2652 Frary 2016	Wrong study design
12. #2097 Hartmann 2019	Wrong study design
13. #1369 Jyotsna 2018	Wrong study design
14. #651 Kiddy 1992	Wrong intervention
15. #2634 Kite 2019	Wrong study design
16. #2132 Lass 2011	Wrong intervention
17. #2181 Li 2021	Nonrandomised and without control group
18. #113 Marsh 2015	Wrong study design
19. #1920 Micić 2003	Wrong intervention
20. #2573 Moran 2006	Wrong study design
21. #1230 Moran 2017	Wrong study design
22. #432 Moran 2011	Wrong study design
23. #2345 Moran 2019	Wrong study design
24. #321 Moran 2006	Wrong intervention
25. #1066 NCT 2019	Study ongoing
26. #1065 NCT 2018	Study ongoing
27. #708 Papakonstantinou 2016	Wrong intervention
28. #742 Pasquali 2011	Wrong study design
29. #1284 Pundir 2019	Wrong study design
30. #1788 Shang 2020	Wrong study design
31. #1331 Song 2020	Wrong intervention
32. #943 Stamets 2004	Wrong intervention
33. #2543 vanDammen 2018	Wrong intervention
34. #462 Varady 2016	Wrong study design
35. #146 Wang 2016	Wrong intervention
36. #2489 Wong 2016	Wrong intervention

## Data Availability

The data that support the findings of this study can be obtained from the corresponding author upon reasonable request.

## Abbreviations

PCOS: Polycystic ovarian syndrome
GLP-1: Glucagon-like peptide 1
DASH: Dietary approach to stop hypertension
TRF: Time-restricted feeding
PRISMA: Preferred Reporting Items for Systematic Reviews and Meta-Analyses
PICO: Population intervention comparison outcome
IGF-1: Insulin-like growth factor 1
HbA1c: Haemoglobin A1c
HOMA-IR: Homeostatic model assessment for insulin resistance
HOMA-B: Homeostatic model assessment beta cell
QUICKI: Quantitative insulin-sensitivity check index
BMI: Body mass index
AUCIns: Area under the curve insulin
AUCGlu: Area under the curve glucose
CRP: C-reactive protein
ALT: Alanine transaminase
NO: Nitric oxide
GSH: Glutathione
SHBG: Sex hormone binding globulin
OGTT: Oral glucose tolerance test
HDLs: High-density lipoproteins
FSH: Follicle stimulating hormone
LH: Luteinizing hormone
E2: Estradiol
TFTs: Thyroid function tests
CINAHL: Cumulative index to nursing and allied health literature
EMBASE: Excerpta Medica Database
WOS: Web of Science
IF: Intermittent fasting
COCP: Combine oral contraceptive pill
PI3K: Phosphoinositide 3-kinase
mTOR: Mammalian target of rapamycin
AMPK: AMP kinase
(c)AMP: (cyclic) Adenosine monophosphate
ATP: Adenosine triphosphate
ERK: Extracellular signal-regulated kinases
MAPK: Mitogen-activated protein kinases
IRS-1: Insulin receptor substrate 1
mRNA: Messenger ribonucleic acid
SIRTs: Sirtuins
FOXOs: Forkhead box Os
PGC-1*α*: Proliferator-activated receptor *γ* coactivator 1*α*
NRF2: Nuclear factor erythroid 2-related factor 2.

## Appendix

### A. Search Strategies

EMBASE (1909 Results)
  1. “ovary polycystic disease”/exp
  2. (“stein leventhal syndrome” OR “cystic ovar^*∗*^” OR “micropolycystic ovar^*∗*^” OR “multiple follicle cyst^*∗*^” OR “ovar^*∗*^ polycystic disease” OR “ovar^*∗*^ polycystic syndrome” OR “polycystic ovarian disease” OR “polycystic ovar^*∗*^” OR “polycystic ovar^*∗*^ disease” OR “polycystic ovar^*∗*^ syndrome” OR “stein cohen leventhal syndrome” OR “stein leventhal disease” OR “syndrome stein leventhal”):ti,ab
  3. #1 OR #2
  4. “insulin”/exp OR “hyperinsulinism”/exp OR “insulin response”/exp
  5. insulin:ti,ab
  6. (“hyperinsulinism” OR “hyperinsulism” OR “hyperinsulinaemia” OR “hyperinsulinema” OR “hyperinsulinemia” OR “insulinaemia” OR “insulinemia” OR “insulin hypoglycaemia” OR “insulin hypoglycemia”):ti,ab
  7. #4 OR #5 OR #6
  8. “fasting”/exp
  9. (Time NEAR/2 restrict^*∗*^ NEAR/2 (Eating OR diet^*∗*^ OR feeding OR fast^*∗*^)):ti,ab
  10. ((“Feeding time^*∗*^” OR diet OR food OR fast^*∗*^) NEAR/3 restrict^*∗*^):ti,ab
  11. (Fasting OR “whole day fast^*∗*^” OR “food tim^*∗*^”):ti,ab
  12. (fast^*∗*^ NEAR/3 diet^*∗*^):ti,ab
  13. ((intermittent^*∗*^ OR alternat^*∗*^ OR modified) NEAR/3 fast^*∗*^):ti,ab
  14. (food NEAR/1 (abstinence or fast^*∗*^)):ti,ab
  15. (Ramadan or Ramadhan):ti,ab
  16. (“16 8 method” OR “5 2 diet”):ti,ab
  17. #8 OR #9 OR #10 OR #11 OR #12 OR #13 OR #14 OR #15 OR #16
  18. #3 AND #7 AND #17
  19. “conference abstract”:it OR “conference report”:it OR letter:it OR editorial:it
  20. #18 NOT #19
Medline (1737)
  1. exp Polycystic Ovary Syndrome/
  2. (stein leventhal syndrome OR cystic ovar^*∗*^ OR micropolycystic ovar^*∗*^ OR multiple follicle cyst^*∗*^ OR ovar^*∗*^ polycystic disease OR ovar^*∗*^ polycystic syndrome OR polycystic ovarian disease OR polycystic ovar^*∗*^ OR polycystic ovar^*∗*^ disease OR polycystic ovar^*∗*^ syndrome OR stein cohen leventhal syndrome OR stein leventhal disease OR syndrome stein leventhal).ti,ab.
  3. or/1-2
  4. exp Insulin/ OR exp Hyperinsulinism/
  5. (insulin OR hyperinsulinism OR hyperinsulism OR hyperinsulinaemia OR hyperinsulinema OR hyperinsulinemia OR insulinaemia OR insulinemia OR insulin hypoglycaemia OR insulin hypoglycemia).ti,ab.
  6. or/4-5
  7. fasting/
  8. (Time adj2 restrict^*∗*^ adj2 (Eating OR diet^*∗*^ OR feeding OR fast^*∗*^)).ti,ab.
  9. ((Feeding time^*∗*^ OR diet OR food OR fast^*∗*^) adj3 restrict^*∗*^).ti,ab.
  10. (Fasting OR whole day fast^*∗*^ OR food tim^*∗*^).ti,ab.
  11. (fast^*∗*^ adj3 diet^*∗*^).ti,ab.
  12. ((intermittent^*∗*^ OR alternat^*∗*^ OR modified) adj3 fast^*∗*^).ti,ab.
  13. (food adj1 (abstinence or fast^*∗*^)).ti,ab.
  14. (Ramadan or Ramadhan).ti,ab.
  15. (16 8 method OR 5 2 diet).ti,ab.
  16. or/7-15
  17. 3 AND 6 AND 16
CINAHL (339)
  1. (MH “Polycystic Ovary Syndrome”)
  2. TI (“stein leventhal syndrome” OR “cystic ovar^*∗*^” OR “micropolycystic ovar^*∗*^” OR “multiple follicle cyst^*∗*^” OR “ovar^*∗*^ polycystic disease” OR “ovar^*∗*^ polycystic syndrome” OR “polycystic ovarian disease” OR “polycystic ovar^*∗*^” OR “polycystic ovar^*∗*^ disease” OR “polycystic ovar^*∗*^ syndrome” OR “stein cohen leventhal syndrome” OR “stein leventhal disease” OR “syndrome stein leventhal”) OR AB (“stein leventhal syndrome” OR “cystic ovar^*∗*^” OR “micropolycystic ovar^*∗*^” OR “multiple follicle cyst^*∗*^” OR “ovar^*∗*^ polycystic disease” OR “ovar^*∗*^ polycystic syndrome” OR “polycystic ovarian disease” OR “polycystic ovar^*∗*^” OR “polycystic ovar^*∗*^ disease” OR “polycystic ovar^*∗*^ syndrome” OR “stein cohen leventhal syndrome” OR “stein leventhal disease” OR “syndrome stein leventhal”)
  3. S1 OR S2
  4. (MH “Insulin+”) OR (MH “Insulin Sensitivity”) OR (MH “Hyperinsulinism+”)
  5. TI (insulin OR “hyperinsulinism” OR “hyperinsulism” OR “hyperinsulinaemia” OR “hyperinsulinema” OR “hyperinsulinemia” OR “insulinaemia” OR “insulinemia” OR “insulin hypoglycaemia” OR “insulin hypoglycemia”) OR AB (insulin OR “hyperinsulinism” OR “hyperinsulism” OR “hyperinsulinaemia” OR “hyperinsulinema” OR “hyperinsulinemia” OR “insulinaemia” OR “insulinemia” OR “insulin hypoglycaemia” OR “insulin hypoglycemia”)
  6. S4 OR S5
  7. (MH “Fasting”)
  8. TI (Time N2 restrict^*∗*^ N2 (Eating OR diet^*∗*^ OR feeding OR fast^*∗*^)) OR AB (Time N2 restrict^*∗*^ N2 (Eating OR diet^*∗*^ OR feeding OR fast^*∗*^))
  9. TI ((“Feeding time^*∗*^” OR diet OR food OR fast^*∗*^) N3 restrict^*∗*^) OR AB ((“Feeding time^*∗*^” OR diet OR food OR fast^*∗*^) N3 restrict^*∗*^)
  10. TI (Fasting OR “whole day fast^*∗*^” OR “food tim^*∗*^”) OR AB (Fasting OR “whole day fast^*∗*^” OR “food tim^*∗*^”)
  11. TI (fast^*∗*^ N3 diet^*∗*^) OR AB (fast^*∗*^ N3 diet^*∗*^)
  12. TI ((intermittent^*∗*^ OR alternat^*∗*^ OR modified) N3 fast^*∗*^) OR AB ((intermittent^*∗*^ OR alternat^*∗*^ OR modified) N3 fast^*∗*^)
  13. TI (food N1 (abstinence or fast^*∗*^)) OR AB (food N1 (abstinence or fast^*∗*^))
  14. TI (Ramadan or Ramadhan) OR AB (Ramadan or Ramadhan)
  15. TI (“16 8 method” OR “5 2 diet”) OR AB (“16 8 method” OR “5 2 diet”)
  16. S7 OR S8 OR S9 OR S10 OR S11 OR S12 OR S13 OR S14 OR S15
  17. S3 AND S6 AND S16
Cochrane Library (268)
  1. [mh “Polycystic Ovary Syndrome”]
  2. (“stein leventhal syndrome” OR “cystic ovar^*∗*^” OR “micropolycystic ovar^*∗*^” OR “multiple follicle cyst^*∗*^” OR “ovar^*∗*^ polycystic disease” OR “ovar^*∗*^ polycystic syndrome” OR “polycystic ovarian disease” OR “polycystic ovar^*∗*^” OR “polycystic ovar^*∗*^ disease” OR “polycystic ovar^*∗*^ syndrome” OR “stein cohen leventhal syndrome” OR “stein leventhal disease” OR “syndrome stein leventhal”):ti,ab,kw
  3. #1 OR #2
  4. [mh “Insulin”] OR [mh “Hyperinsulinism”]
  5. (Insulin OR hyperinsulinism OR hyperinsulism OR hyperinsulinaemia OR hyperinsulinema OR hyperinsulinemia OR insulinaemia OR insulinemia OR “insulin hypoglycaemia” OR “insulin hypoglycaemia”):ti,ab,kw
  6. #4 OR #5
  7. [mh “fasting”]
  8. (Time NEAR/2 restrict^*∗*^ NEAR/2 (Eating OR diet^*∗*^ OR feeding OR fast^*∗*^)):ti,ab,kw
  9. ((“Feeding time^*∗*^” OR diet OR food OR fast^*∗*^) NEAR/3 restrict^*∗*^):ti,ab,kw
  10. (Fasting OR “whole day fast^*∗*^” OR “food tim^*∗*^”):ti,ab,kw
  11. (fast^*∗*^ NEAR/3 diet^*∗*^):ti,ab,kw
  12. ((intermittent^*∗*^ OR alternat^*∗*^ OR modified) NEAR/3 fast^*∗*^):ti,ab,kw
  13. (food NEAR/1 (abstinence or fast^*∗*^)):ti,ab,kw
  14. (Ramadan or Ramadhan):ti,ab,kw
  15. (“16 8 method” OR “5 2 diet”):ti,ab,kw
  16. #7 OR #8 OR #9 OR #10 OR #11 OR #12 OR #13 OR #14 OR #15
  17. #3 AND #6 AND #16
Web of Science (2086)
  1. TS =(“ovary polycystic disease” OR “stein leventhal syndrome” OR “cystic ovar^*∗*^” OR “micropolycystic ovar^*∗*^” OR “multiple follicle cyst^*∗*^” OR “ovar^*∗*^ polycystic disease” OR “ovar^*∗*^ polycystic syndrome” OR “polycystic ovar^*∗*^” OR “polycystic ovar^*∗*^ disease” OR “polycystic ovar^*∗*^ syndrome” OR “stein cohen leventhal syndrome” OR “stein leventhal disease” OR “syndrome stein leventhal”)
  2. TS =(Insulin OR “hyperinsulinism” OR “hyperinsulism” OR “hyperinsulinaemia” OR “hyperinsulinema” OR “hyperinsulinemia” OR “insulinaemia” OR “insulinemia” OR “insulin hypoglycaemia” OR “insulin hypoglycemia”)
  3. TS =((Time NEAR/1 restrict^*∗*^ NEAR/1 (Eating OR diet^*∗*^ OR feeding OR fast^*∗*^)) OR ((“Feeding time^*∗*^” OR diet OR food OR fast^*∗*^) NEAR/3 restrict^*∗*^) OR Fasting OR “whole day fast^*∗*^” OR “food tim^*∗*^” OR (fast^*∗*^ NEAR/3 diet^*∗*^) OR ((intermittent^*∗*^ OR alternat^*∗*^ OR modified) NEAR/3 fast^*∗*^) OR (food NEAR/1 (abstinence or fast^*∗*^)) OR Ramadan or Ramadhan OR “16 8 method” OR “5 2 diet”)
  4. #1 AND #2 AND #3

### B. Excluded Trials

The study ID of the authors and their reason are given in the Table 2.
